# Impact of Scottish Smoke-Free Legislation on Smoking Quit Attempts and Prevalence

**DOI:** 10.1371/journal.pone.0026188

**Published:** 2011-11-16

**Authors:** Daniel F. Mackay, Sally Haw, Jill P. Pell

**Affiliations:** 1 Public Health Unit, University of Glasgow, Glasgow, United Kingdom; 2 Scottish Collaboration on Public Health Research Policy, Edinburgh, United Kingdom; University of Zürich, Switzerland

## Abstract

**Objectives:**

In Scotland, legislation was implemented in March 2006 prohibiting smoking in all wholly or partially enclosed public spaces. We investigated the impact on attempts to quit smoking and smoking prevalence.

**Methods:**

We performed time series models using Box-Jenkins autoregressive integrated moving averages (ARIMA) on monthly data on the gross ingredient cost of all nicotine replacement therapy (NRT) prescribed in Scotland in 2003–2009, and quarterly data on self-reported smoking prevalence between January 1999 and September 2010 from the Scottish Household Survey.

**Results:**

NRT prescription costs were significantly higher than expected over the three months prior to implementation of the legislation. Prescription costs peaked at £1.3 million in March 2006; £292,005.9 (95% CI £260,402.3, £323,609, p<0.001) higher than the monthly norm. Following implementation of the legislation, costs fell exponentially by around 26% per month (95% CI 17%, 35%, p<0.001). Twelve months following implementation, the costs were not significantly different to monthly norms. Smoking prevalence fell by 8.0% overall, from 31.3% in January 1999 to 23.7% in July–September 2010. In the quarter prior to implementation of the legislation, smoking prevalence fell by 1.7% (95% CI 2.4%, 1.0%, p<0.001) more than expected from the underlying trend.

**Conclusions:**

Quit attempts increased in the three months leading up to Scotland's smoke-free legislation, resulting in a fall in smoking prevalence. However, neither has been sustained suggesting the need for additional tobacco control measures and ongoing support.

## Introduction

In Scotland, legislation was implemented at the end of March 2006 prohibiting smoking in all wholly or partially enclosed public spaces. The primary aim of the legislation was to protect non-smokers from exposure to secondhand smoke. A comprehensive evaluation of the impact of the legislation [1] has found that this aim has been largely achieved, with reductions in exposure to secondhand smoke among bar workers [2], the adult general population [3], [4], and children [5], accompanied by improved respiratory health among bar workers [6], and reductions in hospitalisations for childhood asthma [7] and acute coronary syndrome [8]. Studies on the impact of workplace prohibitions suggest that smoking restrictions can also encourage smoking cessation and, thereby, reduce smoking prevalence [9]. It has been suggested that comprehensive legislation covering all public places may have an even greater effect on smoking behaviour [10]. Use of nicotine replacement therapy (NRT) is a good proxy indicator of quit attempts among smokers. A time series analysis of over-the-counter (OTC) NRT sales revealed that Scotland's smoke-free legislation was associated with a short-term increase in OTC sales of NRT [11]. However, OTC sales represent only a small proportion of NRT usage. Also, only 2–3% of quit attempts are successful [12]. Therefore, we analysed the impact of the Scottish legislation on NRT prescriptions and smoking prevalence.

## Methods

### Nicotine replacement therapy

In the United Kingdom, there were a number of changes in NRT provision in 2001 and 2002 including making OTC NRT accessible from non-pharmacy retail outlets and making all NRT products available on NHS prescriptions [13]. Therefore, we analysed NRT data from January 2003 onwards. The Practitioner Services Division of the Scottish National Health Service is responsible for the pricing and processing of all prescriptions that are dispensed outside of hospital, either by community pharmacies or dispensing practices. They also receive information on prescriptions that are issued in Scotland but are dispensed elsewhere in the United Kingdom. We obtained data on the monthly gross ingredient costs of nicotine replacement therapy (NRT) dispensed over a seven year period from January 2003 to December 2009 inclusive; the latter equated to the most recent data available at the time of analysis. The gross ingredient cost is the cost of a drug before deduction of any discounts or special payments made to those prescribing or dispensing the drug. It includes any costs fully or partially reimbursed via prescription charges. The data covered all community-based prescribing sources including general practitioners, the public health service, prescribing nurses, the minor ailment services located in community pharmacies and practice pharmacies. Two sources of NRT prescriptions were not active over the whole study period. NRT was only prescribed by the Minor Ailments Service until the middle of 2007 and by the Public Health Service from 2008. Prescriptions from all sources were included in the overall model for completeness. The data also included prescriptions issued by hospital doctors but dispensed in the community. The data did not cover nicotine replacement therapy purchased over the counter without prescription. These data have already been reported elsewhere [11].

### Smoking prevalence

The Scottish Household Survey (SHS) is funded by the Scottish Government and is a continuous, large-scale social survey of Scottish households and their occupants. The multi-stage sampling is structured to be nationally representative every quarter. Therefore, the data are only available quarterly. Around 26,000 individuals are invited to participate each year and annual response rates are in the range of 66% to 69%, providing information on around 4,000 individuals every quarter. Questionnaires include information on self-reported current smoking status and are completed by fieldworkers using computer aided personal interviewing (CAPI). The Scottish Household Survey provided quarterly data on smoking prevalence over more than 11 years, from January 1999 to September 2010 inclusive; the latter equated to the most recent data available at the time of analysis. Because the smoking prevalence data were only available quarterly, truncation of the study period to January 2003 to December 2009, consistent with NRT data, would have considerably reduced the number of data points and hence statistical power.

### Statistical analyses

Data from the six prescribing sources were aggregated to provide one overall time series. We analysed the overall prescribing data and the smoking prevalence data using Box-Jenkins autoregressive integrated moving average (ARIMA) models [14]. ARIMA was considered preferable to standard interrupted time series models, in that complex time series patterns can be modelled parsimoniously, seasonality within the data is easily handled, past observations are allowed to influence future observations and well established statistical techniques have been developed that allow the impact of interventions on future behaviour to be accurately described and quantified [15]. Individual months vary in the numbers of dispensing days they contain. Therefore, the prescribing series was adjusted for “trading day” effects. We investigated a number of possible models using autocorrelation and partial autocorrelation functions before checking the stationarity properties of both the prescribing and prevalence series using unit root tests [16]. The modelling strategy consisted of initially modelling the whole series to obtain an adequate preliminary model and then modelling and testing the effect of the ban [17]. The Akaike information criterion (AIC) statistic was used to select the most appropriate and parsimonious models prior to testing the intervention hypothesis [18]–[20]. The form of the intervention effect was hypothesised from the time plots of the series. All fitted models were subjected to standard diagnostic checking to ensure that the residuals of the fitted models were not significantly different from those expected from white noise or a random series [21]. We compared the fit from the predicted model and the observed series using the root mean squared error as well as the adjusted R^2^ measure for ease of interpretation. All analyses were undertaken using Stata V11.2 software (STATA Corp, College Station, Texas, URL http://www.stata.com).

This study did not require ethics committee approval. We used secondary data, provided to us as an anonymised and aggregated extract. Identification of individual participants was not possible. Therefore, contacting individual participants to obtain consent was neither necessary nor required.

## Results

### Prescriptions for nicotine replacement therapy

**Figure 1:**
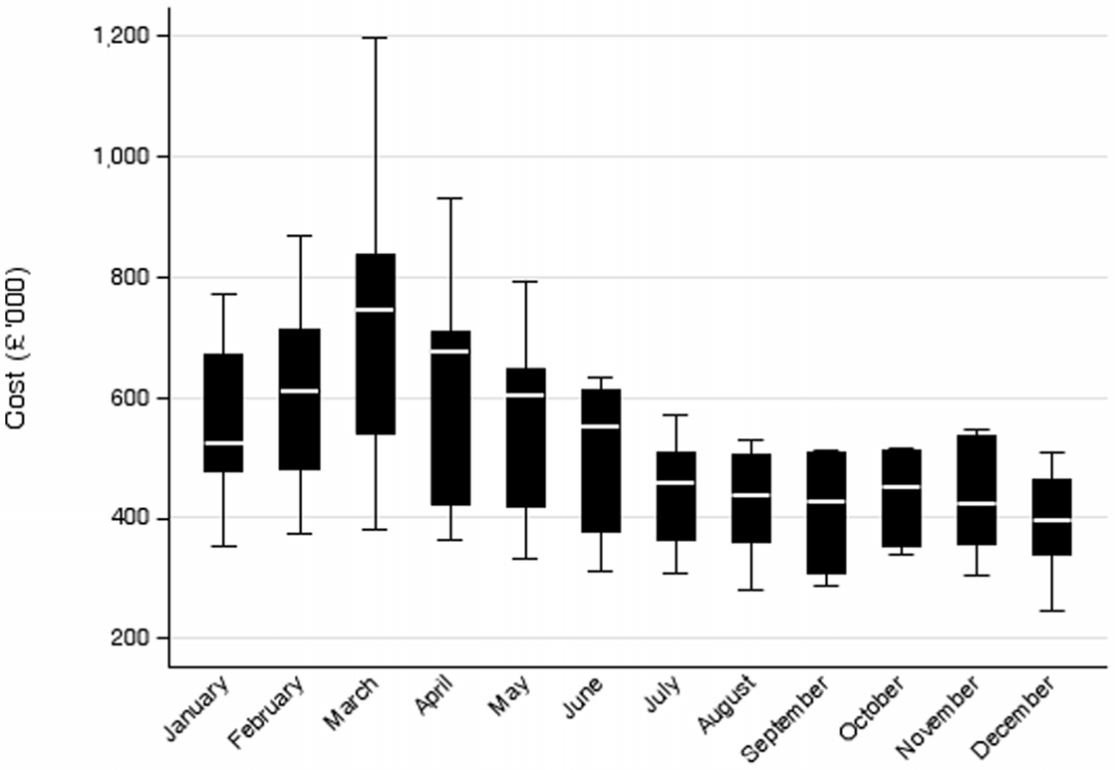
Median and IQR monthly gross ingredient costs of nicotine replacement therapy prescribed in Scotland (2003–2009).

Over the seven year period, the gross ingredient costs of prescribed NRT amounted to £43.3 million. Of these, £34.5 million (79.7%) were due to prescriptions issued by general practitioners, £3.4 million (7.9%) by the public health service (PHS), £2.8 million (6.5%) by prescribing nurses, £1.4 million (3.2%) by the minor ailment services located in community pharmacies, £49,765 (0.1%) by practice pharmacies, £16,932 (0.04%) by hospital doctors and £1.2 (2.8%) million by NHS smoking cessation clinics based in community pharmacies. There was a clear seasonal pattern. Median monthly prescription costs peaked in March, coinciding with No Smoking Day in the United Kingdom, and troughed in December (Figure 1). The median cost of NRT prescriptions issued in March (£747,399) was 88.8% higher than those issued in December (£395,955).

**Figure 2:**
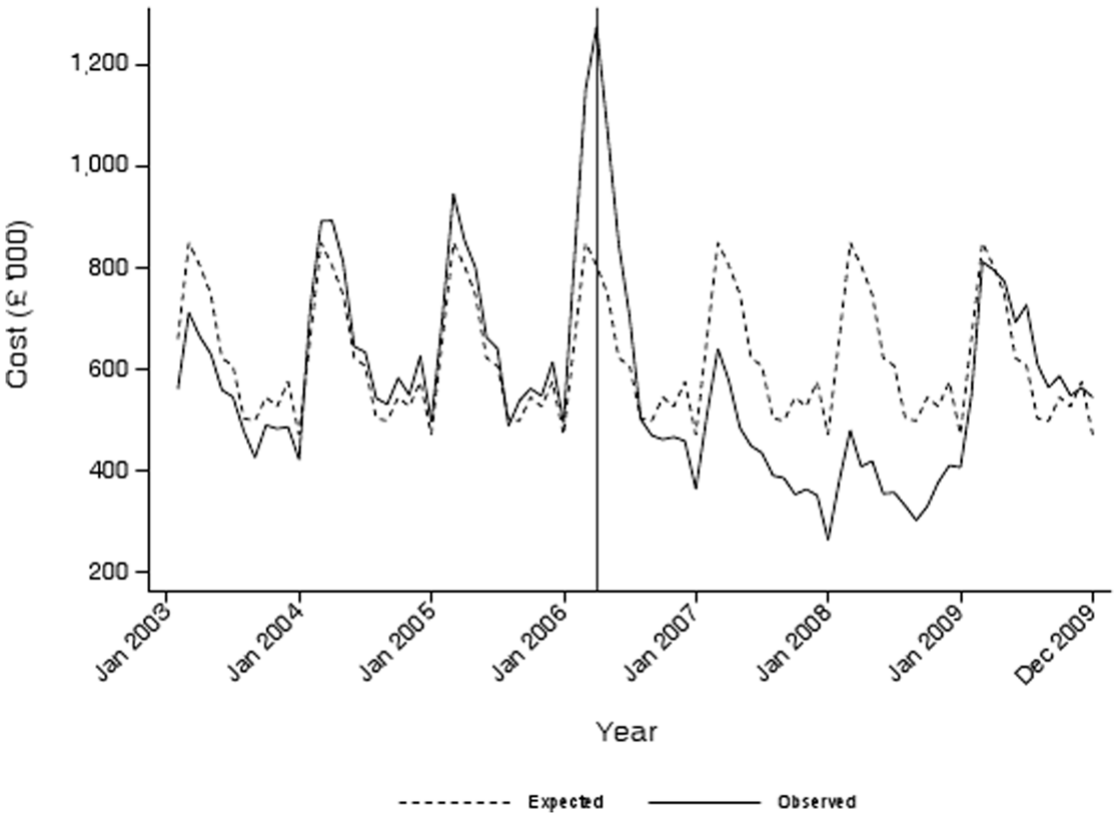
Observed and expected monthly gross ingredient costs of nicotine replacement therapy prescribed in Scotland (2003–2009).

**Figure 3:**
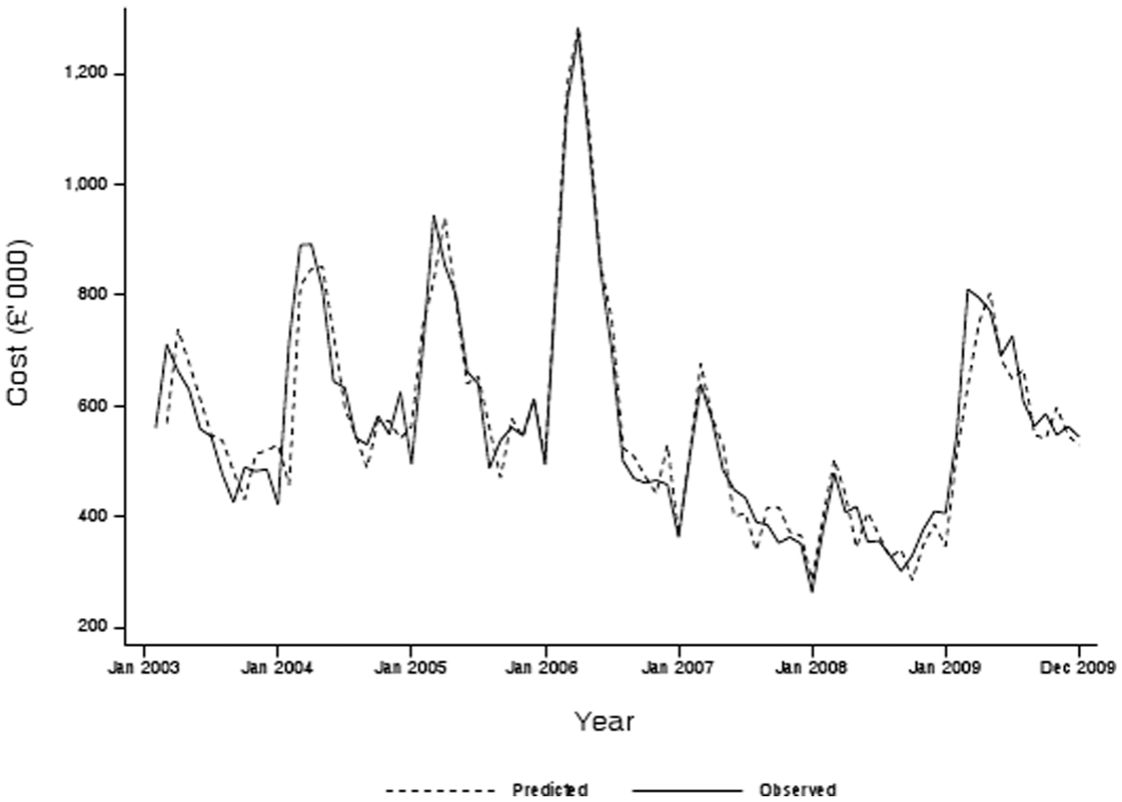
Observed and predicted monthly gross ingredient costs of nicotine replacement therapy prescribed in Scotland (2003–2009).

Figure 2 presents the observed time trends in NRT prescription costs together with the expected time trends. The expected trends are derived from the monthly averages of the pre-ban years (2003–2005) projected beyond 2005. It demonstrates that prescription costs rose dramatically above the pre-ban expected level in March 2006 and then fell after implementation of the legislation before finally reverting to pre-ban expected levels. The pre-legislation rise in prescription costs was not specific to one prescribing source. For the prescribing series the model selected was a multiplicative seasonal ARIMA (SARIMA) with two autoregressive parameters and one autoregressive parameter at the seasonal lag (Table 1). The initial AIC statistic was 2128.9. The Ljung-Box Q statistic for the residuals of the series was 11.43 (p = 0.49) at 12 lags and 18.48 (p = 0.78) at 24 lags indicating that the residuals were “white noise” and the model was a good fit to the series. The adjusted R^2^ for the predicted model fit was 85.5%. To model the introduction of the ban, as well as the anticipatory effect on quit attempts which resulted in two large outliers in January and February of 2006, we included in the SARIMA model dummy variables for January, February and March 2006 as well as a decay parameter, defined as the first lag of the series, from March 2006 onwards. Figure 3 shows the observed and predicted time series using this final intervention model, and demonstrates that the final model was a very good fit for the observed series (adjusted R^2^ = 90.8%) (Table 1). The results of the diagnostic tests applied to the residuals of the model were also reassuring (Ljung-Box Q statistic: 3.00, p = 1.00 at lag 12 and 9.51, p = 1.00 at lag 24). The AIC fell to 2060.4 indicating that the additional parameters improved the fit of the final model (Table 1).

**Table 1:** Time series regression analyses of monthly nicotine replacement therapy gross ingredient costs.

	Initial model		Final model*	
	coefficient (95% CI)	P value	Coefficient (95% CI)	P value
**Monthly, bimonthly and seasonal effects** **				
1 month lag	1.22 (1.03, 1.41)	<0.001	0.30 (0.10, 0.50)	0.003
2 month lag	−0.35 (−0.60, −0.10)	0.006	0.26 (0.05, 0.46)	0.013
12 month lag	0.71 (0.59, 0.83)	<0.001	0.85 (0.74, 0.97)	<0.001
**Legislation effect**				
March 2006 effect	-	-	292,005 (260,402, 323,609)	<0.001
Post March 2006 decay effect	-	-	0.74 (0.65, 0.83)	<0.001
**Regression diagnostics**				
AIC	2,128	-	2,060	-
Q_1_ statistic	0.68	0.41	0.64	0.42
Q_12_ statistic	11.43	0.49	3.00	1.00
Q_24_ statistic	18.48	0.78	9.51	1.00
Adjusted R^2^ (%)	85.5	-	90.8	-
RMSE	72,507	-	58,469	-

*Adjusted for change at March 2006 effect, post March 2006 decay effect, and January/February 2006 peaks.

**Derived from autoregressive moving average model.

CI confidence interval; AIC Akaike Information Criterion; RMSE root mean square error.

NRT prescription costs increased to levels significantly higher than the monthly norms in the three months leading up to implementation of the legislation (Figure 2). NRT prescriptions issued in January and February 2006 were £159,205.3 (95% CI £147,857.7, £170,552.9, p<0.001) and £193,216.6 (95% CI £143,562.1, £242,871.2, p<0.001) respectively higher than their monthly norms. Prescription costs peaked at £1.3 million in March 2006. This was £292,005.9 (95% CI £260,402.3, £323,609, p<0.001) higher than the March norm. Overall prescription costs for 2006 were £7.4 million, 13.8% higher than the 2003–2005 average annual costs of £6.5 million. The estimate of the decay parameter was 0.74 (95% CI 0.65, 0.83, p<0.001). That is, following implementation of the legislation, prescription costs attributable purely to the introduction of the smoking ban fell exponentially by around 26% per month. By 12 months following implementation of the legislation, prescription costs associated with its introduction were not significantly different from the expected 2003 to 2005 pre ban levels, monthly values.

### Smoking prevalence

**Figure 4:**
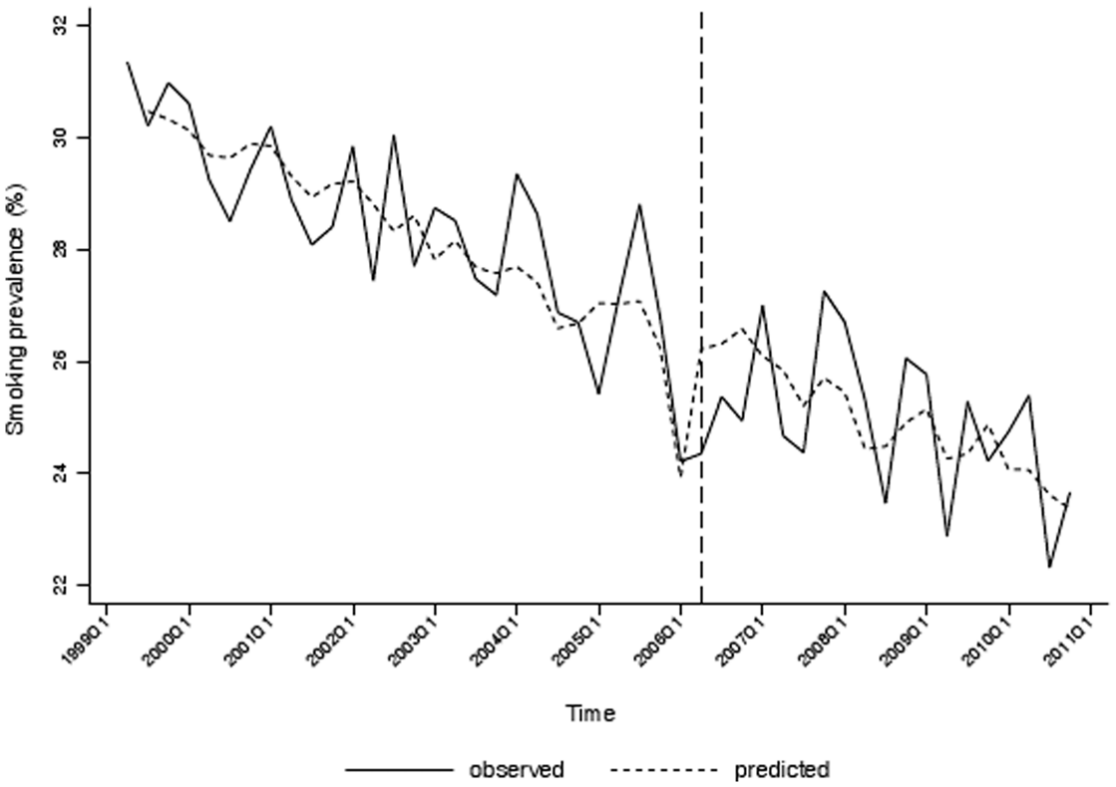
Observed and predicted quarterly smoking prevalence in Scotland (January 1999–September 2010).

The prevalence of self-reported smoking fell by approximately 8.0%, from 31.3% in January–March 1999 to 23.7% in July–September 2010, with a steep decline in prevalence in the quarter preceding implementation of the legislation, followed by a return to levels more consistent with the underlying trend (Figure 4) after approximately one year. The initial model selected for the prevalence data was an ARIMA with one moving average parameter at lag 2 (Table 2). The AIC statistic was 140.3 and the Q statistics for residuals at lags 4 and 8 were 0.44 (p = 0.98) and 5.12 (p = 0.74) respectively. The adjusted R^2^ measure of predicted versus observed fit was 79.4%. The intervention effect of the smoking ban was modelled as being of temporary duration by adding to the model a decay parameter and dummy variable for October–December 2005. The AIC statistic for the final intervention model was 138.5 with the predictive ability of the model improving slightly to an adjusted R^2^ of 79.6%. Q statistics for residual autocorrelation were 0.38 (p = 0.54) at lag one, 1.22 (p = 0.87) at lag 4 and 3.43 (p = 0.90) at lag 8. Figure 4 shows the observed and predicted time series using this model. The model produced a coefficient for the post legislation period dummy variable of −1.70 (95% CI −2.38, −1.02, p<0.001) indicating that in October–December 2005 immediately prior to the introduction of the ban prevalence fell by 1.70% more than expected from the underlying trend. The magnitude of the decay parameter, −0.08, (95% CI −0.38, 0.22) indicates that this effect was short lived with prevalence returning to its long term trend by the last quarter of 2006.

**Table 2:** Time series regression analyses of quarterly smoking prevalence.

	Initial model		Final model*	
	coefficient (95% CI)	P value	coefficient 95% CI	P value
**Six monthly effect** **				
2 quarter moving average	−0.35 (−0.684, −0.007)	0.045	−0.34 (−0.67, −0.10)	0.044
**Legislation effect**				
October/December 2005 effect	-	-	−1.70 (−2.38, −1.02)	<0.001
Post December 2006 decay effect	-	-	−0.08 (−0.39, 0.22)	0.59
**Regression diagnostics**				
AIC	140.3	-	138.5	-
Q_1_ statistic	0.26	0.61	0.38	0.54
Q_4_ statistic	0.44	0.98	1.22	0.87
Q_8_ statistic	5.12	0.74	3.43	0.90
Adjusted R^2^ (%)	79.4	-	79.6	-
RMSE	1.03	-	0.99	-

*Adjusted for change at March 2006 effect, and post March 2006 decay effect.

**Derived from autoregressive moving average model.

CI confidence interval; AIC Akaike Information Criterion; RMSE root mean square error.

## Discussion

### Summary of main findings

In Scotland, smoke-free legislation was associated with a sharp increase in quit attempts, as measured by NRT prescriptions. The increase preceded the legislation by around three months and was accompanied by a 1.7% absolute reduction in smoking prevalence. However, the early benefits have not been sustained, with NRT prescriptions falling to pre-legislation levels within one year of implementation and smoking prevalence also reverting to the underlying trend. This suggests that the prospect of smoke-free legislation accelerated quit attempts and successful quitting among those already planning to quit, but reduced the pool of smokers who were ready to quit and, therefore, the number of quit attempts made in the following months.

### Comparison with other studies

Our findings in relation to NRT prescriptions are consistent with a shorter (2004–2006) time series study of OTC sales of NRT in Scotland which reported that sales increased significantly in the first six months of 2006 but fell thereafter [11]. This study only had information up to one year following the legislation and did not include prescribed NRT. Our study demonstrates that the increase in OTC sales reported in the earlier study cannot be explained by a shift from prescribed NRT to OTC sales, since both increased in the pre-legislation period. Furthermore, our longer time series confirms that, following legislation, NRT use reverted to, and has remained at, pre-legislation levels. In New York, there was also a short-term increase in OTC sales after smoke-free workplace law was introduced, with increases greatest in the week following implementation then declining rapidly afterwards, although pharmacies located in low income areas reported larger and more persistent increases [22]. Scottish adult smokers with high cardiovascular risk who were recruited to an aspirin trial, also reported more quit attempts in the quarter preceding the Scottish legislation, with the mean quit rate increasing from a baseline of 2.3% to 5.1% [23]. Among those who quit, nearly half reported that the legislation had encouraged them to attempt quitting. In a recent study, NRT and bupropion prescribed by general practitioners in England increased in the nine months prior to legislation and fell thereafter [24]. The study did not include NRT prescribed via other routes which, from our results, may account for up to 20% of prescriptions.

The 1.7% absolute reduction in smoking prevalence that followed implementation of the Scottish smoke-free legislation is smaller than reductions previously reported following workplace restrictions. In their meta-analysis, Fichtenberg and Glantz, reported a 3.8% (95% CI 2.8, 4.7) absolute reduction in smoking prevalence, as well as a mean reduction of 3.1 (95% CI 2.4, 3.8) cigarettes smoked per day [9]. Most of the studies included in the review controlled for some potential confounding factors, but may have been subject to selection bias in terms of worker profile and the type of worksite; for example over-representation of health care services. More recently, however, a study of the Italian smoking ban, found that smoking prevalence fell by 1.9% from 26.2% to 24.3%, a figure more commensurate with our findings. The Italian study also reported that the mean number of cigarettes smoked per day fell from 15.4 to 13.9, with the fall most apparent among younger men [25].

### Strengths of our study

Our study has a number of strengths. We used a robust and flexible modelling approach. We had Scotland-wide data from all prescribing sources, including prescriptions issued in Scotland but dispensed elsewhere. We had data over a seven year period, including three years following the legislation, reducing the risk of random variation due to short follow-up. We also had nationally representative quarterly data on smoking prevalence.

### Limitation of our study

The main limitation of our study was the use of overall NRT prescribing as a proxy measure of the numbers of individuals attempting to quit. We did not have access to individual level data on the frequency or duration of NRT use, nor can we identify individuals who attempted to quit on more than one occasion over the seven year period. However, this is unlikely to have introduced a systematic error. Also, we cannot infer whether the reduction in smoking prevalence was due entirely to increased quit attempts or also, in part, to increased success among those who attempted to quit. It is plausible that prohibition of smoking in public places may assist continuation of abstinence.

### Conclusions

The World Health Organisation's Framework Convention on Tobacco Control stipulates that smoke-free legislation should be introduced as part of a comprehensive programme of tobacco control because this approach may be more successful at reducing smoking prevalence [26]. Indeed, in a study in Melbourne, Australia, smoke-free restaurants did not have an independent effect after adjustment for other tobacco control measures [27]. Similarly, in a correlational study of 18 European countries there was no significant association between public place bans and age-sex standardised quit ratio (former smokers : ever smokers) when adjusted for price controls, advertising bans, educational campaigns, health warnings and cessation interventions [28]. In contrast, Grassi et al demonstrated that the effectiveness of smoking cessation interventions was increased following introduction of the Italian smoke-free legislation [29]. The odds of continued smoking at 12 months were reduced by 41% among those undergoing group counselling therapy and by 52% among those receiving bupropion as well as counselling. They demonstrated that the effect was mediated via an increase in motivation. Similarly, a study in the USA demonstrated that comprehensive tobacco control programmes were more effective than price controls used in isolation [30]. In Scotland, smoke-free legislation was one component of a comprehensive tobacco control strategy that had been developed and implemented over the preceding eight years. Scotland's tobacco control strategy is one of the most comprehensive in Europe and includes well developed smoking cessation services and NRT prescribing provided by the National Health Service. In the six months leading up to implementation of the legislation there were two high profile television campaigns. The first ran from September to December 2005 and promoted smoking cessation services and the second ran between January and March 2006 and highlighted the risks associated with exposure to secondhand smoke. During both campaigns calls to Smokeline, the national smoking telephone helpline, greatly exceeded seasonal norms [personal communication: Lesley Cunningham, Essentia Group, Group 2010]. There have been no further campaigns since implementation of the legislation. Our results suggest that the introduction of smoke-free legislation can encourage changes in smoking behaviour. However, if the early benefits are to be sustained in the longer term, legislation needs to be part of a comprehensive programme in which other measures, such as smoking cessation support and media awareness and education campaigns, not only precede legislation but also continue thereafter.
